# OP39 Efficiency Frontier For Relapsing-Remitting Multiple Sclerosis In Brazil: An Approach To System Sustainability

**DOI:** 10.1017/S0266462324001016

**Published:** 2025-01-07

**Authors:** Bruno Barros, Marcelo Correia, Bernardo Tura, Carlos Magliano

## Abstract

**Introduction:**

Several drugs are licensed for the treatment of relapsing-remitting multiple sclerosis (RRMS) in Brazil. The technological horizon of the disease introduces some additional therapies not yet approved in the country. The objective of this study was to establish an efficiency frontier for medicines used for RRMS, to guide the entry of new therapies into the market.

**Methods:**

Primary data on the annualized relapse rate (ARR) and sustained disability progression for six months (CDP6), from 15 different therapies approved in Brazil, were obtained from the literature. Two network meta-analyses (NMA) were conducted comparing multiple treatments based on ARR and CDP6. A Markov model with transition states based on the Expanded Disability Status Scale (EDSS), with an annual cycle and a lifetime time horizon, was built to carry out the cost–utility assessment with an efficiency frontier.

**Results:**

NMA results for ARR and CDP6 were used as inputs for the economic model. In the cost–utility analysis, all strategies were dominated by the lowest-cost medication (teriflunomide) and the most effective one (alemtuzumab). In the deterministic sensitivity analysis, alemtuzumab was the dominant drug in 78 percent of simulations. In probabilistic analysis, most medications had an incremental cost-effectiveness ratio (ICER) below the Brazilian cost-effectiveness threshold in over 90 percent of simulations (approximately USD8,000/quality-adjusted life years [QALY]). An efficiency frontier was established between teriflunomide and alemtuzumab, with an ICER of USD11,141/QALY.

**Conclusions:**

RRMS presents a scenario where several therapies compete for the Brazilian market, but only two of these drugs remained within the established efficiency frontier: alemtuzumab and teriflunomide. For the sustainability of public and private health systems, the efficiency frontier could serve as a cost-effectiveness benchmark, requiring new therapies considering entering the market to adapt to the set parameters.

